# Building from the HIV Response toward Universal Health Coverage

**DOI:** 10.1371/journal.pmed.1002083

**Published:** 2016-08-16

**Authors:** Jonathan Jay, Kent Buse, Marielle Hart, David Wilson, Robert Marten, Scott Kellerman, Morolake Odetoyinbo, Jonathan D. Quick, Timothy Evans, Peter Piot, Mark Dybul, Agnes Binagwaho

**Affiliations:** 1 Harvard T. H. Chan School of Public Health, Boston, Massachusetts, United States of America; 2 UNAIDS, Geneva, Switzerland; 3 International HIV/AIDS Alliance/STOP AIDS NOW!, Washington, D.C., United States of America; 4 World Bank Group, Washington, D.C., United States of America; 5 Rockefeller Foundation, New York, New York, United States of America; 6 Management Sciences for Health, Medford, Massachusetts, United States of America; 7 Positive Action for Treatment Access, Lagos, Nigeria; 8 Harvard Medical School, Boston, Massachusetts, United States of America; 9 London School of Hygiene and Tropical Medicine, London, United Kingdom; 10 Global Fund to Fight AIDS, TB and Malaria, Geneva, Switzerland; 11 Geisel School of Medicine, Hanover, New Hampshire, United States of America; 12 University of Global Health Equity, Kigali, Rwanda

## Abstract

Jonathan Jay and colleagues draw lessons from the the global HIV response that could help guide the universal health coverage movement.

**Summary Points**

- Universal health coverage (UHC) has gained prominence as a global health priority. The UHC movement aims to increase access to quality, needed health services while reducing financial hardship from health spending, particularly in low- and middle-income countries.
- As a policy agenda, UHC has been identified primarily with prepayment and risk-pooling programs. While financing policies provide important benefits, increasing access to health services will require broader reforms.
- For lessons, the UHC movement should look to the global HIV response, which has confronted many of the same barriers to access in weak health systems. Considerable success on HIV has resulted from innovative approaches that UHC efforts can build upon, in areas including governance, financing, service delivery, political mobilization, accountability, and human rights.
- UHC and HIV efforts must capitalize on potential synergies, especially in settings with a high HIV burden and major resource limitations.

## Introduction

Universal health coverage (UHC) has gained prominence as a global health objective. United Nations (UN) member states endorsed UHC in a 2012 resolution [1] and adopted it as a Sustainable Development Goal (SDG) target in 2015 [2].

These global agreements conceptualize UHC as ensuring all people’s access to the health services they need, with sufficient quality to be effective, while protecting against the financial risk of out-of-pocket health spending. Global health agencies have proposed monitoring countries’ UHC progress by the proportion of the population whose financial protection and health service needs are met, at prespecified levels [3]. Countries would determine which services they measure, except for a set of core global indicators.

Contemporary formulations of UHC dispense with the idea that countries can “achieve” UHC simply by enrolling a large proportion of the population in financing programs. Rather than nominal coverage—the formal entitlement to services—the accepted approach implies effective coverage, in which people actually receive all the services they need and experience better health as a result [4]. Recognizing that effective coverage gaps exist even in the highest-performing health systems, this approach considers UHC an aspirational end state, pursued as “a direction rather than a destination” [4].

To operationalize the current understanding of UHC through public policy, normative guidance must keep pace. To date, health financing reforms have received primary attention as drivers of UHC [5]. These reforms are central: government-led prepayment and risk-pooling mechanisms can significantly reduce out-of-pocket spending, catastrophic health spending, and impoverishment [6]. They are associated with increased utilization of health services, especially among the poor [7].

Improving access to services, however, requires UHC efforts to lift many more barriers. Discrimination, poor quality of care, low capacity, and other resource limitations undermine health service provision in many settings. Lifting these barriers requires significant political commitment and, often, vastly improved performance from government and other service providers.

The best example of global expansion in needed health services is the HIV response. This effort continues to face setbacks and shortcomings, including high rates of new infection among young women and marginalized groups, discriminatory laws, and approximately 22 million people in need of antiretroviral therapy (ART) [8]. However, it has achieved a 35% reduction of new HIV infections since 2000 and delivered ART to 15 million people [8]—three-quarters in Africa, where skeptics once doubted large-scale ART coverage was possible. As a result, AIDS-related deaths have declined by 42% since peaking in 2004 [8].

Scaling up HIV treatment and prevention services worldwide involved transformative change [9]. Following early years of confusion, misinformation, and inaction, the movement pioneered models of political activism and mobilization, reshaped institutions and norms for health governance, advanced human rights, tackled social and economic determinants of health, dramatically increased external and domestic financing for health, and revitalized critical aspects of health systems in high-burden countries [10,11].

As the UHC movement confronts major deficits in access, such as 400 million people lacking basic health services [3], we propose looking to the HIV response for lessons. Below, we recommend approaches from the HIV response that UHC efforts can adapt and repurpose. We focus on low- and middle-income countries (LMICs) because the HIV response has been concentrated there and because UHC strategies may be most catalytic in these settings [12]. We also offer guidance for aligning programs in settings with a high HIV burden.

## Recommendations

### Governance: Include the Public and Civil Society

The HIV response has established paradigms for including citizens in health governance at all levels, with unprecedented participation through mechanisms such as the Global Fund Board, Country Coordinating Mechanisms, and the Joint UN Programme on HIV and AIDS (UNAIDS) Programme Coordinating Board. Community groups and networks, often operating outside the formal health system, have strengthened community systems to extend services to marginalized and stigmatized populations. Alongside ministries and parliaments, civil society organizations engage in formal arrangements for monitoring HIV programs [13].

UHC proponents should conceptualize UHC reforms as a partnership between government and the public [14]. Inclusive governance brings the public into processes such as (a) priority-setting for benefits packages and other resource allocation; (b) oversight for health services, including quality and adherence to copayment policies; and (c) monitoring performance against stated goals [15]. The HIV experience suggests that improving public involvement and accountability is a wise step to address common UHC challenges such as a gap between de jure and de facto benefits packages [16] and could help accelerate coverage for high-priority populations and interventions.

### Financing: Situate Pooled Financing Mechanisms within Broader Reforms

As discussed above, prepayment and risk-pooling arrangements can improve equity, increase utilization, and reduce impoverishment; they should not, however, represent the full extent of new budgeting towards UHC. As the HIV response encountered, where health systems are weak, new funds cannot go towards purchasing alone. Scaling up HIV services required financial investments in the health workforce, facilities, the pharmaceutical supply chain, and other needs [11]. Many investments simultaneously advanced broader health aims [17]: for example, in Ethiopia, external HIV funding contributed to the Health Extension Program, which has recruited, trained, and supported over 35,000 community health workers providing primary health care, including HIV services, in rural settings [18].

New UHC financing offers potential synergies with HIV financing: when UHC reforms invest in health systems, HIV budgets can focus on HIV-specific interventions. One option is to fund HIV-related health services through the broader pool devoted to UHC. HIV treatment, however, represents a large share of health spending in LMICs with a high HIV burden; it might be necessary to protect the pool, at least in its early stages, with supplemental external or domestic financing earmarked for HIV. Otherwise, high demand for HIV treatment could make the pool fiscally unsound. Ghana, for example, funds ART outside its national health insurance pool [19]; Brazil [20] and Thailand [21] did so until fairly recently.

Funding all HIV services through broader financing pools may bring efficiency gains. A well-governed pool can become an appropriate recipient for health assistance grants, as in Rwanda. Countries growing towards middle-income status and away from external financing could potentially self-finance HIV services through increased allocations to UHC financing pools, as long as necessary public health programs retain funding.

### Service Delivery: Build on Effective Platforms without Compromising Access

#### Build on effective platforms

In countries with large HIV programs, UHC efforts must interface with those programs to maximize common platforms and avoid inefficiency and duplication. Integration of HIV programs with other interventions is not new, particularly with services for directly related coinfections such as tuberculosis, blood-borne infections, maternal and child health, and sexual and reproductive health. Increasingly, HIV programs incorporate interventions for chronic diseases and conditions, particularly those whose risk is increased by HIV infection and those that require similar delivery platforms [22].

Scaling up needed services will require further integration [22]. Efforts should be guided by a pragmatic principle: build on whatever works best. In Zambia, for example, delivering antimalarial drugs through the ART supply chain system was found significantly more effective and less costly than a system designed de novo [23].

#### Preserve focused service delivery programs for marginalized groups

Many HIV prevention and treatment efforts focus on marginalized populations who face barriers (mostly nonfinancial) to health system access. Key populations have typically been men who have sex with men, transgender people, people who use drugs, and sex workers and their clients; they can include young women, migrants, ethnic minorities, and prisoners, among others, in different contexts [24]. Ensuring effective coverage of services among these groups should be considered essential to a pro-UHC approach; however, less-targeted UHC strategies, such as insurance, are not structured to achieve this aim. Elevated HIV risk and uncertain access to public services require tailored interventions [25]. While focused programs are often controversial and rarely a political priority, they are absolutely critical for human rights, sexual and reproductive rights, and public health.

At the same time, prepooling programs should be as inclusive as possible. Thailand, for example, adapted to the transition away from Global Fund support partly by creating mechanisms for undocumented migrants to obtain national health insurance [26].

#### Decentralize service delivery and engage communities

For increasing access in LMIC settings, the HIV response has demonstrated the effectiveness and responsiveness of decentralized, community-based primary care platforms, as in Rwanda (see Box 1 below). Involving communities themselves and building bridges to community institutions is key to create demand, ensure quality, and facilitate utilization among rural populations and informal sector workers. Additionally, empowering women and community-based nongovernmental organizations (NGOs) to assist in service delivery can complement necessary public services, create employment, and significantly improve access for marginalized populations [27,28].

Health workforce gaps represent a major obstacle to UHC efforts [29]. The HIV response, facing the same challenge, experimented with task shifting at multiple levels, generating significant evidence on what works, what does not, and how to assess those programs [30]. UHC research should investigate opportunities for smart task shifting, especially for priority interventions.

**Box 1. Coordinating HIV and UHC Investments in Rwanda**

Between 2000 and 2013, Rwanda achieved dramatic progress in effective health coverage, including for HIV. AIDS-related mortality dropped approximately 80%, and UNAIDS targets for universal prevention of mother-to-child transmission (PMTCT) and ART coverage were achieved [31]. Meanwhile, national health insurance plan coverage surpassed 90% [32].

While this success is well documented, the interdependence of the HIV and UHC agendas in Rwanda deserves attention. Both are central to the Rwandan government’s Vision 2020 strategy, implemented since 2000. This cross-sectoral strategy has aligned contributions from multiple ministries and through public–private partnership and civil society participation.

Rwanda has coordinated programs to increase synergies between HIV and UHC, including through its management of development assistance funding. The ministry of health has prioritized integrated, community-based platforms and evidence-based practice: HIV-specific interventions were integrated into efforts to strengthen primary care and provide all Rwandans more equitable access and more comprehensive health services [41]. Supply chains established to deliver drugs for HIV were used to deliver all types of products. Information systems designed to track HIV treatments were modified to track all treatments. Health workers in maternity wards are trained through PMTCT programs and deliver infants irrespective of maternal HIV status.

Governance approaches have also been inclusive. Rwanda’s HIV national response formalized civil society’s governance role through reserved seats on the board of the former National AIDS Control Commission (2001–2010) and the ongoing Global Fund Country Coordinating Mechanism. Within UHC efforts, the biannual Joint Health Sector Review convenes government, development partners, and civil society to assess all national health programs. Ten civil society representatives join this review on behalf of a range of key populations and constituencies.

### Political & Social Mobilization: Develop Narratives and Demand Action

The HIV response succeeded not just because its agenda was technically sound but because it tapped larger narratives and built political demand. The public must demand UHC.

Although respondents in LMIC surveys prioritize quality health care [33], grassroots activism for comprehensive, affordable health services has been limited. Such activism can help create pressure for UHC reforms: in Thailand, for example, civil society organizers collected thousands of signatures supporting equitable health care access prior to enactment of the reforms [34].

Communications campaigns can tap into larger political narratives. AIDS campaigns argued that it is unfair that some people should die, and others survive, based simply on income, place of birth, sexual orientation, or gender identity [10]. UHC can connect to related movements concerning justice across geographical boundaries (for example, global North–South), socioeconomic justice within LMICs, intellectual property regimes, labor policies and social protection initiatives, and the women’s movement, as well as many others.

UHC advocates can also draw from AIDS campaigns by connecting UHC to economic growth and national security. They can cite evidence on the economic benefits of health reform, including the estimate that health system investments will generate 9- to 20-fold returns in LMICs by 2035 [35].

Weak, unjust health systems pose security concerns. HIV advocates established political momentum by highlighting societal and economic threats, resulting in the first UN Security Council resolution on a health issue, in 2000 [36]. Ebola was the second, in 2014 [37]; UHC proponents have argued that UHC reforms can improve resilience against infectious disease threats like Ebola, through stronger service delivery systems and financing arrangements and increased public trust [38]. Targeted capacities for controlling outbreaks, such as disease surveillance and response, can be packaged with health financing reforms, as in Mexico’s 2003 legislation [39].

In the HIV response, calls to action were not limited to the community level. Champions such as former Presidents Festus Mogae of Botswana and Olusegun Obasanjo of Nigeria and former UN Secretary-General Kofi Annan led major national and international efforts. Similarly, top political leaders have been critical to successful UHC legislation. The UHC movement should recruit them actively. Advancing UHC not only is an obligation but may provide political benefits [40].

### Targets and Monitoring: Ensure Equity for Marginalized Groups

Whether led by communities or politicians, emphasizing human rights or socioeconomic security, a focus of the HIV response has been clear, bold ambition with messages and arguments that resonate from the “elevator speech” to in-depth analysis. For UHC, robust data—extensive, credible, and disaggregated by subgroup—are necessary to drive ambition and accountability [41]. Data can empower decision makers in ministries and parliaments, along with civil society as watchdogs. UHC monitoring should quantify not just inputs, such as individuals enrolled, but also process indicators, such as the utilization and quality of key services, and disease-specific health outcomes, including for HIV [15].

Moreover, monitoring should measure equity across multiple dimensions of vulnerability and marginalization. While UHC proponents have suggested disaggregating by income, gender, and geography [3], we recommend countries strengthen their health information infrastructure so policy makers can also track access by age, disability status, ethnicity, sexual orientation, and other locally relevant dimensions of health equity.

### Social Determinants of Health: Lift Access Barriers through Rights-Based Policies and Programs

The health system alone cannot achieve UHC [42]: as the HIV response has illustrated, many barriers to access lie beyond the health sector. For example, discriminatory laws and policies, including criminalization, can severely undermine access to health services for marginalized groups [11]. The AIDS movement has argued for inclusive and protective laws and policies and decriminalization, to hold governments accountable for creating an enabling environment for people at particular risk.

Such multisectoral actions should be part of a UHC reform agenda cutting across government divisions—a “health in all policies” approach [43]. Additionally, civil society and communities can identify, and help eliminate, barriers to access; empowering civil society and communities in governance, therefore, may help mitigate adverse social determinants of health.

## Conclusions

We have argued that UHC programs cannot concern themselves exclusively with financial measures—they must address all of the barriers to effective coverage and coordinate with existing health initiatives, including the HIV response.

This approach could help accelerate global HIV efforts [44]. While the HIV response has achieved enormous progress, there remain critical shortfalls in prevention and treatment services and the struggle for social inclusion and equity. An historic effort has only taken us partway, leaving an urgent need for sustainable, forward-looking strategies.

UHC reforms provide a vehicle for governments to increase their health investments. Alongside continued support from international partners, these much-needed investments could bolster the financial sustainability of HIV programs by strengthening health systems, promoting economic growth, and, over time, reducing reliance on external financing. UHC reforms could also expand access to the other health services required by the millions of people who are already accessing ART.

These potential synergies demand further collaboration. With 169 SDG targets—13 in health alone [2]—sharing lessons and resources will be vital. The UHC and HIV movements could provide a model for coordinated action in the next era of global health.

## Abbreviations

ART: antiretroviral therapy
LMICs: low- and middle-income countries
NGO: nongovernmental organization
PMTCT: prevention of mother-to-child transmission
SDG: Sustainable Development Goal
UHC: universal health coverage
UN: United Nations
UNAIDS: Joint United Nations Programme on HIV and AIDS
